# Mapping Cyber Bot Behaviors: Understanding Payload Patterns in Honeypot Traffic

**DOI:** 10.3390/s26010011

**Published:** 2025-12-19

**Authors:** Shiyu Wang, Cheng Tu, Yunyi Zhang, Min Zhang, Pengfei Xue

**Affiliations:** 1 College of Electronic Engineering, National University of Defense Technology, Hefei 230037, China; wangshiyu@nudt.edu.cn (S.W.); tucheng@nudt.edu.cn (C.T.); zhangyyzyy@nudt.edu.cn (Y.Z.); 2 Institute for Network Sciences and Cyberspace, Tsinghua University, Beijing 100190, China

**Keywords:** internet measurement, network security, honeypot, traffic payload, machine learning, large-scale payload analysis

## Abstract

Cyber bots have become prevalent across the Internet ecosystem, making behavioral understanding essential for threat intelligence. Since bot behaviors are encoded in traffic payloads that blend with normal traffic, honeypot sensors are widely adopted for capture and analysis. However, previous works face adaptation challenges when analyzing large-scale, diverse payloads from evolving bot techniques. In this paper, we conduct an 11-month measurement study to map cyber bot behaviors through payload pattern analysis in honeypot traffic. We propose TrafficPrint, a pattern extraction framework to enable adaptable analysis of diverse honeypot payloads. TrafficPrint combines representation learning with clustering to automatically extract human-understandable payload patterns without requiring protocol-specific expertise. Our globally distributed honeypot sensors collected 21.5 M application-layer payloads. Starting from only 168 K labeled payloads (0.8% of data), TrafficPrint extracted 296 patterns that automatically labeled 83.57% of previously unknown payloads. Our pattern-based analysis reveals actionable threat intelligence: 82% of patterns employ semi-customized structures balancing automation with targeted modifications; 13% contain distinctive identity markers enabling threat actor attribution, including CENSYS’s unique signature; and bots exploit techniques like masquerading as crawlers, embedding commands in brute-force attacks, and using base64 encoding for detection evasion.

## 1. Introduction

Cyber bots are automated programs that carry out unsolicited activities, including vulnerability scanning/exploitation, unauthorized login attempts, and pre-attack reconnaissance behaviors, etc. Examples include tools like Masscan [1], Hydra [2], Metasploit [3], and various application clients. The unsolicited activities of cyber bots present potential security threats to the modern Internet. First, their targets are extensive, spanning a wide range of industries and their associated services [4,5]. Second, their low cost and high operational efficiency have driven widespread proliferation, with cyber bot traffic accounting for 49.6% of global Internet traffic in 2023 [5]. Moreover, the emerging cyberspace search engines such as Shodan [6] and Censys [7] utilize cyber bots to index asset information, inadvertently expanding the attack surface for adversaries [8,9]. Consequently, a comprehensive understanding and analysis of cyber bot behavior are essential for strengthening network security.

Analyzing the payload patterns of packets transmitted by cyber bots provides the most direct and insightful understanding of their behavior [10,11,12]. For example, a pattern containing specific command keywords (e.g., wget http://x.x.x.x/a.sh) may indicate a download-and-execute behavior. These unsolicited activity payloads are commonly captured in the wild using trap-based monitoring sensors, such as network telescopes and honeypots [13]. Traditionally, network telescopes have been non-interactive, also known as darknets. Recently, researchers have enhanced these systems with the ability to complete TCP handshakes, making them capable of capturing one payload per TCP connection from cyber bots [14,15]. However, these systems are still unable to receive multi-interaction payloads, thereby limiting the depth of payload information they can analyze. On the contrary, honeypots are capable of capturing multi-interaction traffic [16], providing valuable insights into core requests, password attempts, and commands executed by cyber bots [17,18,19,20]. Honeypot payload analysis has thus established itself as the primary research paradigm for understanding cyber bot behaviors.

Nonetheless, studies [18,21,22] have reported coverage rates ranging from 1% to 60% when analyzing large-scale, diverse honeypot traffic payloads [18,21,22,23,24,25,26,27,28], and this wide variation reflects fundamental adaptation limitations: the rapid evolution of cyber bots constantly introduces new payloads that existing rule-based or narrowly trained models struggle to cover. For rule-based methods [18,21,23,24,27], these approaches leverage manually constructed rules to recognize specific patterns in honeypot payloads (i.e., label the payloads), serving as the basis for subsequent analysis. The quality of analysis depends on the comprehensiveness of their existing rules, but maintaining up-to-date rules requires substantial manual effort as new patterns emerge, making large-scale deployment challenging in practice. To address this challenge, several machine learning-based methods have been proposed, such as learning from payload features to automate behavioral analysis [22,25]. However, these methods face significant limitations in adapting to diverse payload analysis. Protocol-specific feature engineering approaches [22] require designing separate features and models for different protocols, while clustering-based methods [25] produce abstract group labels that lack semantic meaning and necessitate extensive manual inspection to understand behaviors. Overall, in existing studies, the proportion of payloads that can be effectively analyzed remains constrained by predefined rules, protocol-specific features, or abstract labels, hindering a comprehensive understanding of payload information in cyber bot behavior analysis.

**Our work.** To overcome the above limitations, we propose TrafficPrint, a framework that automates the extraction of reusable payload patterns across heterogeneous protocols, provides semantically meaningful labels for newly observed cyber bot activities, and systematically analyzes their behaviors in the wild. The key insight is that cyber bots, being automated programs, inevitably produce structurally and semantically similar payloads when executing the same technique repeatedly. TrafficPrint exploits this characteristic through a three-stage framework: (1) leveraging pre-trained language models to create semantic embeddings of payloads without protocol-specific feature engineering, (2) clustering these embeddings to identify groups of payloads sharing similar technical characteristics, and (3) extracting common substrings within each cluster as human-understandable patterns. This combination of representation learning and pattern mining enables TrafficPrint to handle heterogeneous protocols while maintaining the interpretability essential for security analysis.

**Figure 1:**
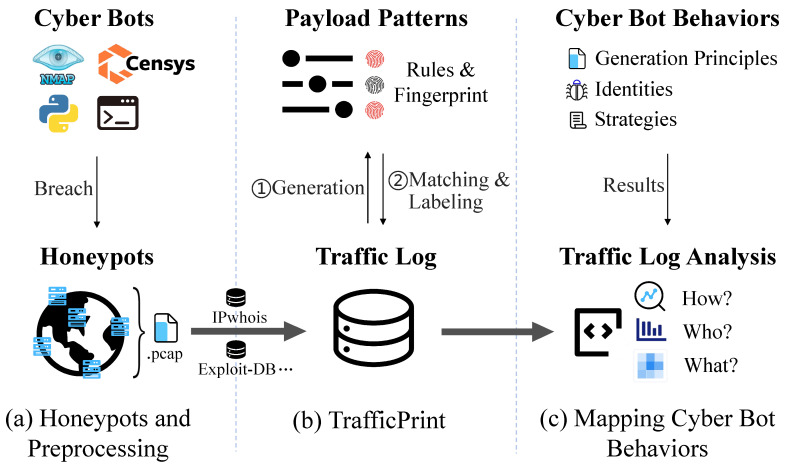
High-level workflow diagram of our work.

We perform a systematic measurement study of cyber bot behaviors through large-scale and diverse payload pattern analysis. Figure 1 shows the workflow of our study. To collect comprehensive cyber bot traffic, we deploy 50 honeypots globally across 21 different services, capturing 71 M traffic logs over 11 months (August 2023 to June 2024), of which 21.5 M are application-layer payloads for pattern extraction. Using only 168K rule-labeled [29,30] payloads (0.8% of payloads) to bootstrap, TrafficPrint extracted 296 interpretable patterns that automatically label 83.57% of previously unlabeled traffic. These patterns transform millions of raw payloads into a structured technical repertoire, enabling systematic mapping of cyber bot behaviors.

**Our findings.** With the measurements and analysis of payload patterns produced by cyber bots, we map their behaviors across three dimensions:

How are they generated? We reveal the *generation principles of cyber bot behaviors from the perspective of payload structure, including fully customized, semi-custom, and default principles* (Section 7.1). Fully customized payloads (11% of patterns) do not conform to any well-known protocols, created by specific organizations for particular objectives. Semi-custom payloads (82% of patterns) follow known protocol formats but contain tailored elements, such as targets and parameters. Default payloads (7% of patterns) come from official protocol examples, enabling adversaries to directly construct cyber bots for service availability testing. Beyond structural principles, 54% of the payload patterns exhibit temporal regularities, indicating time-based coordination in cyber bot behaviors.

Who is behind them? We find that 13% of the payload patterns reveal a general rule, where *payloads can explicitly and implicitly convey identity* (Section 7.2). Explicitly, some payloads declare identity through content, including Censys, exposure tools like Masscan, or academic organizations stating research intentions. Implicitly, identity is not directly stated but can be inferred from unique customization styles or behavioral preferences, making the payload itself a recognizable signature of its origin. For example, only CENSYS employs the payload pattern *1\r$11\rNONEXISTENT\n.

What strategies are reflected in them? By integrating multi-dimensional information, we validate that *the payloads reflect cyber bots’ strategic preferences across different behaviors* (Section 7.3) identifying behaviors such as service scanning (78.14%), web crawling (0.39%), brute-force attacks (19.98%), vulnerability scanning (0.62%), and exploitation (0.86%). We find that cyber bots may disguise themselves as legitimate crawlers to evade detection, embed standard commands within brute-force attacks to bypass account locking mechanisms, or use base64 encoding in exploit commands to circumvent security filters.

**Our contributions.** The main contributions are as follows:

- We propose TrafficPrint, a framework that automatically extracts human-understandable payload patterns from large-scale, diverse honeypot payloads without requiring protocol-specific feature engineering. (Section 5).
- We apply TrafficPrint to 21.5 M honeypot payloads, successfully extracting 296 interpretable patterns that automatically label 83.57% of previously unknown traffic, creating a new set of payload rules for cyber bot analysis (Section 6.2).
- We perform a systematic pattern-based analysis of cyber bot behaviors in the wild, revealing generation principles across different patterns, and demonstrating how payload patterns can elucidate the identities, techniques, and evasion strategies employed by cyber bots (Section 7).

## 2. Background & Related Work

This section provides the foundation for understanding our approach to cyber bot behavior characterization. We first define cyber bots and their role in cybersecurity (Section 2.1), establishing the scope and significance of the problem. We then review existing methods for analyzing honeypot traffic (Section 2.2), critically examining their strengths and limitations in extracting behavioral patterns from large-scale, diverse traffic. Finally, we discuss language models and their applications in cybersecurity (Section 2.3), highlighting how contextual representations address challenges in payload understanding.

### 2.1. Cyber Bots and Cybersecurity

We define cyber bots as automated programs identified by IP addresses, which target remote hosts to perform unsolicited activities. These activities include: (1) sending specially crafted payloads to applications, and (2) collecting information such as services and webpages from the response packets sent by the remote hosts, or performing actions such as privilege escalation and exploitation. While cyber bots serve legitimate purposes in security research and Internet measurement, they can also be leveraged by malicious actors for attacks. The dual-use nature of these tools makes understanding their behavioral patterns essential for both security researchers and network defenders. Extensive research has investigated behaviors such as crawling [21], scanning [15], and brute-force attacks [17,31] to uncover their strategies.

### 2.2. Honeypot Traffic Analysis

Given the need to understand cyber bot behaviors, honeypot sensors have become essential tools for capturing their activities. Despite the ability of honeypots to collect rich data, the value of this data hinges on the analytical methods used to extract meaningful behavioral insights. Among these, the payload information, capturing the content fields of network traffic, plays a crucial role in detecting behavior patterns [12] and identifying devices [32]. Compared to other aspects (e.g., scanning frequency, IP address), payloads exhibit greater stability amid evolving cyber bot techniques. Such payload patterns correspond to the third layer of the Pyramid of Pain [10], making them particularly disruptive to adversaries.

Researchers have explored various approaches to extract actionable threat intelligence from honeypot traffic, with many methods fundamentally relying on payload, often implemented through rule-based techniques [27] or traffic-feature clustering. (1) Rule-based solutions: Li et al. [21] embedded TLS signatures into honeypot systems to identify the true carriers of web bots and analyzed their behaviors using HTTP formatting-based payload rules. Favale et al. [24] simulated 15 service types in an unused campus network and used rule-based analysis of brute-force login payloads to study attacks from a horizontal perspective. Munteanu et al. [18] simulated legitimate SSH/Telnet services and categorized honeypot sessions into five types based on specific payload rules derived from SSH/Telnet commands. (2) Traffic-feature clustering solutions: Griffioen et al. [23] exposed honeypots implementing open and unauthenticated protocols like NTP, DNS, and SSDP to attract amplification attacks, grouping similar attack payloads for analysis. Shamsi et al. [26] deployed multiple open-source honeypots and applied a consensus clustering method combined with independent payload clustering results to classify attacker IPs, identifying IPs controlled by a single operator.

*While the above traditional solutions provide some understanding of cyber bot behaviors, they face adaptation limitations as they rely on predefined patterns (rules or features). As cyber bot techniques continuously evolve and diversify, this reliance leads to delayed coverage and incomplete insight into cyber bot behaviors at scale.* To address these limitations, recent studies have adopted representation learning for deeper analysis of cyber bot behaviors. Barbosa et al. [25] applied NLP techniques to group attacker shell interaction payloads, uncovering underlying intentions and operational patterns. Sheng et al. [22] captured scanning traffic using a distributed ICS honeynet and proposed a representation method based on ICS payload features, along with a self-adaptive multi-class classification model to improve the accuracy and adaptability of scanner group identification.

*Although these approaches combine behavior analysis with advanced learning models, they focus on specific protocol domains and produce abstract group labels that cannot be directly translated into actionable rules for practical threat analysis.* Consequently, existing approaches, whether relying on predefined patterns (rules or features) or producing abstract labels, exhibit poor adaptability to evolving bot behaviors, limiting analytical coverage to only 1–60% [18,21,22] of honeypot traffic and hindering comprehensive behavioral mapping. Our work addresses these limitations by focusing on mapping cyber bot behaviors through payload patterns in large-scale, cross-protocol honeypot traffic, aiming to consistently produce interpretable patterns that can represent the majority of emerging behaviors. To achieve this goal, we propose a framework that integrates the strengths of representation learning (through language models that capture semantic similarities across diverse protocols), traffic clustering, and rule-based techniques. This framework enables adaptation to the evolving nature of cyber bots, ensures reliable labeling, and provides actionable systematic insights for mapping cyber bot behaviors in the wild.

### 2.3. Language Models and Cybersecurity

Recent progress in contextual language modeling has increasingly influenced cybersecurity research. Payloads and logs can be viewed as sequential data whose semantics depend heavily on the surrounding content. Models such as BERT [33] and RoBERTa [34] learn bidirectional contextual representations that reflect these dependencies, and recent works have further explored semi-supervised or contrastive objectives to enhance such contextual encodings [35]. This allows them to capture semantic similarity between syntactically different payloads and to distinguish similar byte patterns that serve different functions in different locations. Such properties are essential for understanding heterogeneous traffic in honeypot environments.

Traditional representation methods such as TF-IDF, Bag of Words, Word2Vec [36], or FastText [37] assign fixed embeddings to tokens and struggle with unseen or rare byte combinations. This limitation is well-documented in prior cybersecurity work on traffic representation [38]. In contrast, BERT-like models generate context-dependent encodings that naturally generalize to previously unseen inputs. Several studies have already applied BERT or RoBERTa to network payloads and logs, including payload classification [39], log parsing and anomaly detection [40,41,42]. These results show that contextual models consistently outperform domain-specific embeddings that rely on manual tokenization or protocol heuristics.

Motivated by these works, we adopt RoBERTa [34] as the backbone of our payload encoder. RoBERTa is trained on larger corpora and does not rely on sentence-level structure, which aligns well with the fragmented and boundary-free nature of honeypot payloads. Its Byte Pair Encoding tokenizer also accommodates mixed textual and hexadecimal content more effectively than traditional embedding methods.

## 3. Honeypot Dataset

### 3.1. Honeypot Infrastructure

We deploy 50 honeypot sensors across 8 geographic regions to collect comprehensive cyber bot traffic for pattern analysis. Each honeypot runs on a virtual machine configured with 2 CPU cores and 2 GB RAM, with the deployment sharing 1 GB/s network bandwidth. Individual honeypots host 3–7 services selected from a pool of 21 available protocols, creating varied service combinations such as HTTP, FTP, SSH, HTTP, MySQL, RTSP, and HTTP, Telnet, MongoDB.

**Figure 2:**
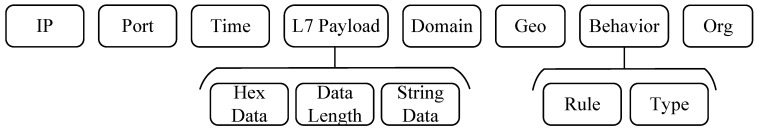
Traffic log field structure for each action.

Our honeypots are equipped with built-in data processing capabilities that automatically transform captured packets into structured traffic logs during collection. Each log entry represents a semantically meaningful action, which may be a connection establishment, a connection termination, or an application-layer payload transmission. In particular, each payload is recorded as a separate action. Each log entry contains the fields shown in Figure 2, including IP addresses, ports, timestamps, and application-layer (L7) payload data when applicable.

**Payload Preprocessing.** We extract L7 payload data and store it in multiple formats to support analysis approaches. As shown in Figure 2, each payload is represented as hexadecimal data, ASCII string data, and payload length, enabling both binary-level and text-based pattern analysis.

**Behavioral Labeling.** Given that specific rules can only cover a limited subset of all possible cyber bot behaviors ($|{⋃}_{r\in R}\mathrm{Behaviors}\left(r\right)|\ll |\mathrm{All}\;\mathrm{Cyber}\;\mathrm{Bot}\;\mathrm{Behaviors}|$), we organize behaviors into five categories C={service scanning, web crawling, brute-force, vulnerability scanning, exploitation} that can comprehensively cover cyber bot behaviors ($|{⋃}_{c\in C}\mathrm{Behaviors}\left(c\right)|\approx |\mathrm{All}\;\mathrm{Cyber}\;\mathrm{Bot}\;\mathrm{Behaviors}|$), as defined in Table 1.

To provide ground truth for these categories, we label L7 payloads using public rules from Exploit-DB [29] and Nuclei-Templates [30] for exploitation behaviors, payload features from [21] for web crawling, protocol-specific authentication patterns for brute-force attacks, and characteristic signatures for service scanning and vulnerability scanning. This creates a mapping f:R→C consistent with Table 1, where each matched rule generates a behavior label containing both the rule identifier and corresponding category, as shown in Figure 2.

**Geographic and Attribution Enrichment.** To understand the sources and actors behind the traffic, we enrich each log entry with geographic and organizational information (corresponding to the Geo, Domain and Org fields in Figure 2). We utilize the Geo database to obtain geographical location information associated with each IP address. Additionally, we check the adversary’s IP using WHOIS and reverse DNS. If WHOIS shows an organization name (e.g., Censys), or reverse DNS returns a domain name linked to a known organization, we treat the IP as non-anonymous. Otherwise, it is considered anonymous. For anonymous IPs, we search our honeypot’s IP in multiple cyberspace search engines and collect matching results with timestamps and ports. These results are then correlated with local traffic to determine whether the anonymous IP originated from a specific search engine (i.e., organization).

### 3.2. Dataset Characteristics

From August 2023 to June 2024, our honeypots recorded 71,047,534 application-level actions from 149,136 unique IP addresses. The actions consist of connection establishment actions (29.1 M), L7 payload transmission actions (21.5 M), and connection termination actions (20.4 M). While the 21.5 M payload actions form our primary dataset for pattern extraction, we analyze the broader context across all 71 M traffic logs to understand dataset characteristics.

**Figure 3:**
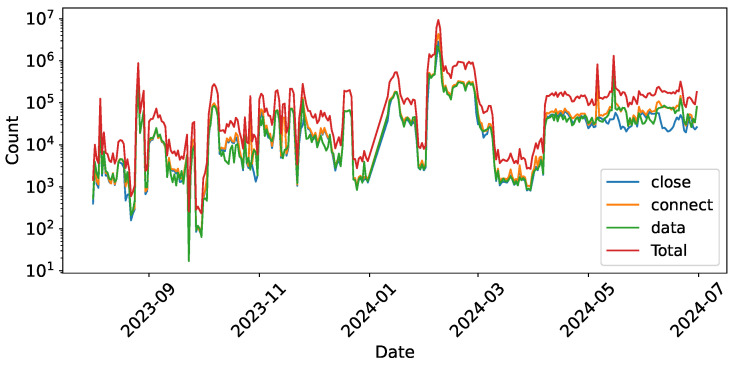
Daily action summary (log scale).

**Connection Distribution**. Figure 3 shows the traffic volume across these stages, highlighting a noticeable discrepancy between connection establishment and termination counts, particularly in May and June 2024. Our statistical results reveal that 48.76% of IP addresses display an imbalance between the number of connect and close actions. Analyzing the causes of this phenomenonwe inspected the raw packets corresponding to these actions. We find that among these IPs with asymmetric connection behaviors, 91% exhibit TCP packets with the SYN flag set to 1 and the ACK flag set to 0. This pattern is consistent with well-known cyber bot behaviors such as SYN scans or SYN flood attacks [23,43]. For the remaining 9%, the asymmetry appears to result from rapid connection establishment or extended connection durations that trigger our honeypot’s protective mechanisms. When client connections exceed limits (10 connections) or a single connection surpasses duration thresholds (10 s), the honeypot enforces queue mechanisms and sends RST packets to forcibly close existing connections. Overall, the prevalence of these connection imbalances across nearly half of the observed IP addresses indicates that our dataset contains cyber bot behaviors, including scanning and reconnaissance activities.

**Figure 4:**
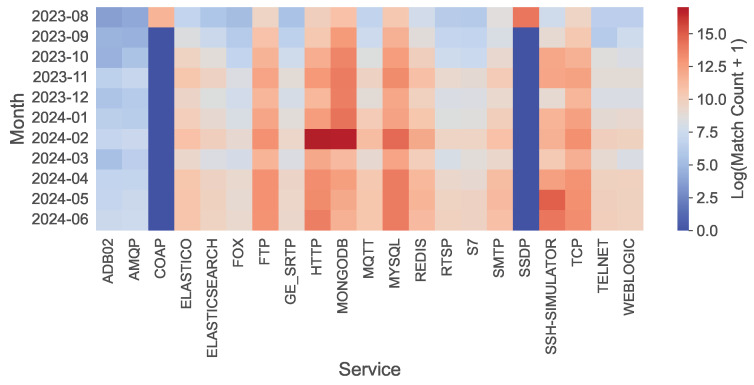
Heatmap showing the distribution of data volume received by 21 services over a period of 11 months (ln scale). The *x*-axis label “TCP” refers to a bare TCP port with no application-layer service deployed.

**Service Diversity.** Protocol distribution demonstrates comprehensive bot targeting (Figure 4). HTTP and MongoDB peak in February 2024, while FTP, MQTT, MySQL, Redis, and RTSP maintain persistent traffic. Volumes increase over time for most services, indicating sustained bot interest, and only COAP and SSDP were decommissioned due to overwhelming targeting. Across the 21.5 million L7 payloads, substantial heterogeneity is observed (Table 2), with no single protocol dominating the dataset. HTTP accounts for 24.37%, MongoDB for 22.36%, and the top-5 protocols collectively represent 65.22%, while the remaining 34.78% is distributed across 16 additional protocols including Telnet, SMTP, RDP, and others. This distribution confirms that the dataset encompasses a broad and varied spectrum of L7 protocols, providing quantitative evidence of its heterogeneity.

**Behavioral Diversity.** Honeypots label 168,451 samples from the 21.5 M collected payloads, achieving only 0.8% coverage. The low labeling coverage indicates substantial behavioral diversity in our dataset, with the vast majority of cyber bot activities exhibiting patterns not captured by these established rule sets. Analysis of the unlabeled data reveals diverse payload structures, novel protocol interactions, and previously unseen patterns, suggesting our dataset contains a rich spectrum of both known and potentially novel cyber bot behaviors.

Despite the low overall coverage, the 168,451 labeled samples provide valuable ground truth for model training. The distribution across behavioral categories is: service scanning (69,247 samples), web crawling (36,457 samples), brute-force (31,866 samples), vulnerability scanning (6853 samples), and exploitation (24,028 samples). For model training and evaluation, we use these coarse-grained behavioral categories from C as supervision signals, which provide sufficient semantic diversity for effective domain adaptation. This approach avoids the overfitting issues that arise from training on fine-grained rules, many of which have extremely limited sample sizes with some appearing in fewer than 5 samples, making them unsuitable for reliable model training. However, in practical deployment scenarios, security analysts require fine-grained behavioral patterns corresponding to specific rules from R for precise behavior identification and analysis. This motivates our framework design: we train on category-level labels to learn robust behavioral representations, then leverage these learned embeddings through unsupervised clustering to automatically discover and extract fine-grained payload patterns that correspond to specific behavioral rules.

**Figure 5:**
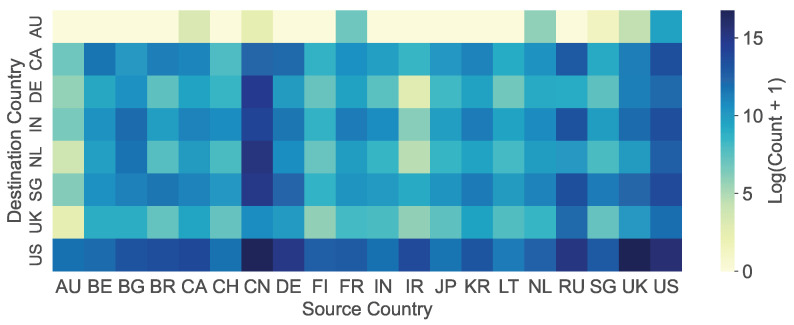
Heatmap of traffic received by honeypots from the top 20 source regions (ln scale).

**Geographic and Source Diversity**. Figure 5 shows the heatmap of traffic received by honeypots from different regions, highlighting the top 20 sources. These top 20 source regions include the locations where our honeypots are deployed, confirming that honeypots predominantly collect traffic from their immediate geographic vicinity. Furthermore, cyberspace search engines tend to use IP addresses from their home countries for scanning activities [9], and several regions hosting popular search engines appear among the top 20 source regions. This correlation indicates that our honeypots capture cyber bots originating from cyberspace search engines. In fact, we identify that some cyber bots belong to cyberspace search engines: 65 IPs belong to Shodan, 337 IPs belong to Censys, 11 IPs belong to ZoomEye [44], and 7001 IPs belong to BinaryEdge [45].

## 4. Ethics

In this study, all cyber bot traffic analyzed was collected and handled within secure, controlled environments to ensure that no personally identifiable information (PII) was exposed. The honeypots were deployed to anonymize network details, and specific configurations and locations were withheld to prevent potential exploitation. Sensitive information, such as IP addresses, was anonymized in accordance with legal and ethical standards, ensuring the privacy and security of legitimate network participants while enabling meaningful analysis of cyber bot behaviors and strategies.

The honeypots were configured across 21 protocols covering a broad range of service categories, including web services, database systems, industrial control protocols, IoT services, remote access services, file-transfer services, real-time streaming, and general network services. This selection reflects the protocols most frequently targeted during the observation period, though it may introduce a form of selection bias because unmonitored services could exhibit different behavior patterns. In addition, some sophisticated bots may attempt to detect honeypots; to reduce this risk, we periodically update the device fingerprints presented by the honeypots to avoid being identified by major Internet-wide scanners. Although the monitored protocols and the 11-month observation window cannot fully capture behavior patterns that evolve over longer timescales or appear on unmonitored services, the observations derived from this deployment still provide meaningful insight into cyber bot activities within this representative operational setting.

## 5. Proposed Framework: TrafficPrint

### 5.1. Overview

This section presents TrafficPrint, an effective automated framework for extracting human-understandable payload patterns as rules from cyber bot traffic. The adaptability of TrafficPrint primarily lies in addressing two challenges: One is that cyber bots often target a wide range of network services, including unknown or less-documented ones [22], making protocol-specific feature extraction insufficient for capturing the full range of their behaviors. The other is that automatically extracted payload patterns must be human-understandable rather than merely machine-readable [22,25], to enable analysts to more efficiently validate them, integrate them into analysis workflows, and map them to cyber bot behaviors in the wild.

**Figure 6:**
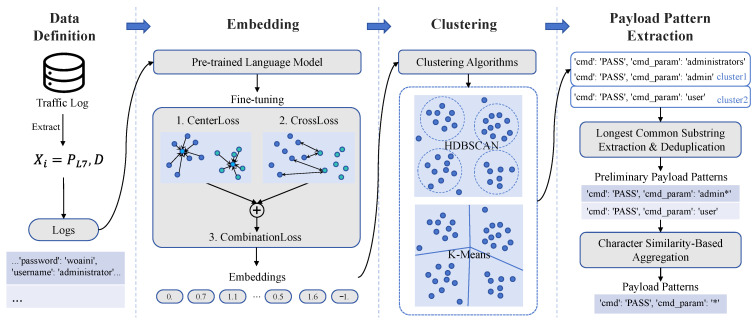
Components and operating principles of TrafficPrint. ‘*‘ is used as a placeholder for variable regions in the patterns.

As shown in Figure 6, TrafficPrint addresses these challenges through two key design principles: (1) Universality. It directly takes logs containing raw payload information collected by honeypots as input to a pre-trained language model, without requiring protocol-specific expert knowledge, enabling payloads from arbitrary services to be processed uniformly. We fine-tune a pre-trained model on a small number of labeled payloads to construct a semantic embedding space tailored to large-scale and diverse payload data. (2) Explainability. It combines representation learning, clustering, and string extraction algorithms to extract human-understandable payload patterns as rules. By embedding unlabeled payloads into a semantic space and clustering them, TrafficPrint identifies payload groups, significantly reducing the scope of rule generation. Within each cluster, common substrings are extracted as payload patterns, which support subsequent large-scale automatic labeling and annotating cyber bot behaviors.

### 5.2. Input Data Definition

Cyber bots tend to generate large volumes of similar payloads, resulting in highly consistent traffic patterns. This consistency is particularly evident in common cyber bot behaviors such as brute-force attacks and network scanning, where most payload content remains static while only target content varies dynamically. This inherent similarity allows cyber bots to exhibit distinct characteristics in network traffic analysis, often resembling patterns found in natural language. Although our log data, as shown in Figure 2, includes various attributes, considering the strong semantic relevance and independence from service-specific structures, we focus primarily on the payload content itself when generating payload patterns. Additionally, adversaries often reuse carefully designed cyber bots to streamline operations and reduce costs, making domain information valuable for grouping similar traffic. We construct the input for each payload action by concatenating the L7 payload-related information and the domain, represented as $P_{L7}=\left\{\mathrm{hex}\;\mathrm{data},\mathrm{data}\;\mathrm{length},\mathrm{string}\;\mathrm{data}\right\}$ and *D* respectively. This results in:

(1) $$X_{i}=P_{L7},D$$

where the comma “,” denotes the concatenation operation. The corresponding label $y_{i}$ represents the behavior category from C rather than the specific rule from R, as shown in Figure 2. As discussed in Section 3, using behavior types as labels better ensures embedding adaptability across diverse payload patterns.

### 5.3. Embedding

Next, we convert honeypot traffic data into semantic embeddings. To achieve this, we utilize the RoBERTa pre-trained model [34], an enhanced version of BERT, which offers several advantages for large-scale and diverse honeypot payloads: (1) traditional NLP methods such as TF-IDF, Bag of Words (BoW), and Word2Vec encounter difficulties in representing unseen words [38], which conflicts with our task. In contrast, RoBERTa, leveraging Byte Pair Encoding (BPE), efficiently handles a combination of text and hex data in honeypot traffic logs. Importantly, BPE tokenization treats all payloads uniformly as byte sequences regardless of their protocol origin, ensuring that protocol diversity does not fragment the representation space; (2) RoBERTa eliminates BERT’s Next Sentence Prediction (NSP) task, which is designed for structured text with clear sentence boundaries, and instead focuses on robust masked language modeling with larger training corpora. This is particularly beneficial for our traffic logs, which are unstructured payloads without sentence boundaries, making NSP irrelevant while the enhanced sequential modeling capability of RoBERTa becomes more valuable; (3) Compared with fully opaque black-box embedding models, the attention-based architecture of RoBERTa enables a lightweight post hoc inspection of which byte-level regions of the payload receive higher emphasis during representation learning, which is valuable for downstream behavior analysis. These three properties are critical for effective representation learning on honeypot payloads, as further validated by the experiments in Section 6.1.

Building on this, we apply layer-wise fine-tuning to adapt the model and obtain an embedding space better suited for the traffic domain. To improve the generalization ability of the fine-tuned model and ensure that the generated embeddings effectively reflect behavioral similarity rather than superficial protocol features, we introduce a combined loss function that integrates Center Loss and Cross-Entropy Loss.

The combined loss function integrates both Center Loss and Cross-Entropy Loss to improve the model’s clustering and classification performance. As shown in Figure 6, Center Loss encourages samples of the same class to cluster around their centroids, enhancing the grouping of similar cyber bot behaviors. Cross-Entropy Loss ensures accurate classification, distinguishing between different cyber bot behaviors. We define the final combined loss function as:  

(2) $$L_{\mathrm{combined}}=\left(1-\lambda \right)\frac{1}{N}\sum_{i=1}^{N}{∥f_{i}-c_{y_{i}}∥}^{2}-\lambda \frac{1}{N}\sum_{i=1}^{N}\sum_{c=1}^{C}y_{i,c}\log\left(p_{i,c}\right)$$

where *N* represents the number of samples, $f_{i}$ is the feature vector of sample *i*, $c_{y_{i}}$ is the centroid of class $y_{i}$, $y_{i,c}$ denotes the true label for sample *i* and class *c*, $p_{i,c}$ is the predicted probability for class *c*, and λ is a hyperparameter balancing the two loss terms.

This combined loss function enhances the model’s ability to both cluster similar samples and accurately classify them, leading to improved robustness and generalization in the analysis of honeypot traffic.

### 5.4. Clustering

After obtaining the embedded representations of the traffic logs, TrafficPrint performs clustering to group traffic with common patterns together. The selection of appropriate clustering algorithms is critical, as honeypot traffic exhibits distinct characteristics (Section 3) that constrain algorithmic choices. First, the data shows highly imbalanced cluster distributions: certain behavioral patterns such as scanning activities generate substantial volumes of similar payloads, while others like targeted exploitation produce fewer but distinct samples. Second, real-world honeypot data inevitably contains irregular and incomplete payloads that do not correspond to coherent behavioral patterns, necessitating robust noise handling [46]. Third, the number of distinct behavioral patterns is unknown a priori and varies significantly across different deployment environments, making fixed-K algorithms unsuitable for unsupervised discovery.

Given these requirements, we select HDBSCAN [47] and K-Means [48] for their complementary strengths. Partition-based methods (K-Means, GMM) assume relatively uniform cluster sizes and require predefined cluster counts, poorly suited for imbalanced data with unknown categories. Hierarchical methods [49] force every sample into some cluster, causing noise to propagate through the dendrogram and distort discovered patterns. Graph-based approaches [50,51] can discover clusters without specifying *K*, but require computing and storing full pairwise similarity matrices, making their $O(n^{2})$ memory complexity computationally prohibitive for large-scale datasets. In contrast, HDBSCAN addresses all three requirements: it naturally accommodates varying cluster densities through density connectivity, explicitly identifies and excludes outliers rather than forcing them into clusters, and automatically determines cluster numbers without user specification. Furthermore, HDBSCAN achieves O(nlogn) complexity through efficient hierarchical density estimation, making it scalable to large honeypot datasets. HDBSCAN requires specification of two key hyperparameters: min_cluster_size and min_samples, which control the minimum cluster size and density threshold for core points, respectively. We set these values based on the statistical properties of our dataset (detailed in Section 6.2). While HDBSCAN is well-suited for unsupervised pattern discovery, we retain K-Means for validation purposes. When ground truth labels exist during model development, K-Means provides a controlled framework to assess whether our embedding space produces semantically meaningful clusters given the known number of categories.

Thus, TrafficPrint employs both algorithms strategically: K-Means is used for structural validation and model evaluation on labeled data with known behavior types C, providing guidance for understanding the embedding space quality. HDBSCAN is applied to unlabeled data to automatically discover fine-grained clusters corresponding to specific behavior rules R, where the number of distinct payload patterns is unknown.

### 5.5. Payload Pattern Extraction

While clustering groups semantically similar payloads together, security analysts require interpretable patterns to understand the underlying cyber bot techniques and generate actionable rules. To extract common structural elements from clustered payloads and represent them as human-understandable strings, this module employs a two-stage pattern extraction process, as shown in Figure 6.

**Stage 1: Preliminary Pattern Extraction.** For each traffic cluster $S_{i}\in S$, we extract preliminary patterns by finding the longest common substrings between payload pairs. The algorithm identifies contiguous matching blocks B between strings and replaces variable regions with placeholders (denoted as Glob). Each preliminary pattern thus consists of one or more common substrings and zero or more placeholders(e.g., GET */login, POST /user/*/data). Since these preliminary patterns are extracted from payloads already clustered in a semantic embedding space using a pre-trained RoBERTa model, the approach is robust to noise and minor variations. Although it only captures contiguous substrings, longest common substring provides high interpretability for analysts, while more complex NLP-based pattern mining methods may require additional computation and can produce patterns less human-understandable. For clusters with multiple payloads, we select the pattern with maximum coverage as the cluster representative. After deduplication, this generates a set of preliminary patterns $P_{prelim}$.

**Stage 2: Pattern Generalization.** An ideal payload pattern should be generalizable, capturing the core structure shared across similar payloads while covering a broad set of samples. To achieve this, we compute a similarity matrix *M* where $M_{ij}=\mathrm{SequenceRatio}\left(p_{i},p_{j}\right)$ quantifies structural similarity between patterns. Agglomerative clustering with distance threshold (1−θ) groups similar patterns, where θ is the similarity threshold controlling how closely patterns must match to be clustered together. Each resulting cluster g∈G produces a generalized pattern capturing similar structures.

Algorithm 1 formalizes this complete extraction pipeline. These patterns serve multiple roles: they represent millions of payloads as concise string patterns, label unseen behaviors to expand label coverage, and ultimately enable scalable and in-depth cyber bot behavior analysis.
**Algorithm 1:** Payload Pattern Extraction Process.
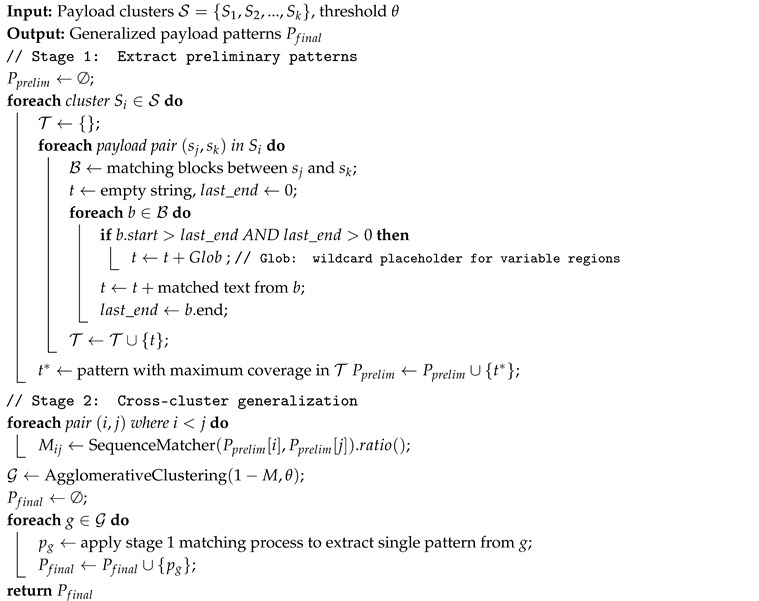



## 6. Framework Evaluation and Application

Before conducting behavioral analysis, this section first evaluates the effectiveness of TrafficPrint’s core components, then applies it to extract payload patterns from our honeypot traffic logs, generating more comprehensive labeled datasets.

### 6.1. Validation on Labeled Data

Based on the 168,451 labeled samples from behavioral labeling (Section 3), we randomly split each behavioral category into stratified training (80%), validation (10%), and testing (10%) sets, ensuring representation across all categories despite their highly imbalanced distribution (Section 3.2). We fine-tune the pre-trained RoBERTa-base model on the training set. We evaluate five embedding approaches on these datasets: (1) Word2Vec trained on the training set as a domain-specific baseline (vector size 768, window size 5, min count 1), (2) pre-trained RoBERTa-base without fine-tuning, and three RoBERTa fine-tuning methods ( Section 5.3) using (3) center loss (λ=0), (4) cross-entropy loss (λ=1), and (5) combined loss. For the combined loss, we sweep λ∈{0.1, 0.3, 0.5, 0.7, 0.9} on the validation set and select the best-performing value, which is λ=0.5 for the main experiments. All fine-tuning uses identical configurations based on recommended ranges for transformer fine-tuning [33,34] and validated on our dataset: learning rate 0.00005, batch size 32, and 10 epochs. We refer to these approaches as Word2Vec, RoBERTa-base, RoBERTa-Center, RoBERTa-Cross, and RoBERTa-Combined in our results and visualizations.

**Fine-tuning enables domain-specific embeddings for traffic logs**. For quantitative evaluation, we use K-means clustering for downstream evaluation on the testing set. To ensure stable and reproducible results, we employ K-means++ initialization [52] and run the algorithm 10 times with different random seeds, selecting the solution with minimum inertia. We report four standard clustering metrics: Purity [53], measuring the proportion of correctly assigned samples in each cluster; $F_{1}$ score [53], reflecting the precision-recall balance; Fowlkes-Mallows Index (FMI) [54], capturing the pairwise consistency between predicted and true labels; and Adjusted Rand Index (ARI) [55], quantifying the similarity between clustering results and ground truth while correcting for chance. Table 3 shows the performance hierarchy across different approaches. Word2Vec, trained specifically on our traffic log domain, significantly outperforms the pre-trained RoBERTa-Base, which suffers from the domain gap between general natural language and network traffic data. However, all fine-tuned RoBERTa variants surpass both baselines, with RoBERTa-Combined achieving the best performance across all metrics.

Due to the near-perfect scores of RoBERTa-Combined on the full training data, we further examine the reasonableness of these results by conducting sensitivity analyses on the combined loss weight λ and the size of the training data. For this analysis, we vary only the training set size, using 20% and 50% subsets of the training data from the main experiments, while keeping the validation and testing sets identical (Table 3). The validation set is not used to select λ in this analysis. Table 4 reports clustering performance across different values of λ. We observe that performance generally improves as λ increases from 0 to 0.5, remains high for λ between 0.5 and 0.7, and slightly declines at the extremes (λ=0 or 1). This pattern is consistent for both smaller (20% of full training set) and larger (50% of full training set) training sets, indicating that the method is robust to moderate variations in λ and training data size. These results confirm that fine-tuning produces stable and reasonable domain-specific embeddings, enabling RoBERTa-Combined to outperform both pre-trained models and traditional domain-specific methods.

**Figure 7:**
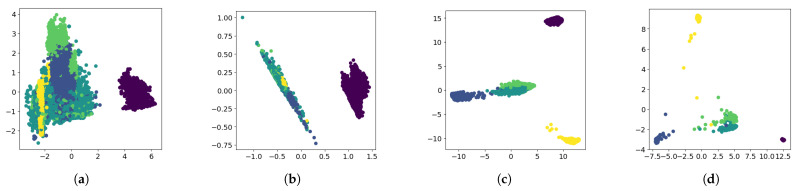
PCA visualization of traffic log embeddings. Points of the same color belong to the same class. Fine-tuning improves embedding structure and separability. (**a**) RoBERTa-Base, where embeddings of different colors are mixed; (**b**) RoBERTa-Center, distances within each color are reduced but classes are still mixed; (**c**) RoBERTa-Cross, different colors are distinguishable but partially overlapping; (**d**) RoBERTa-Combined, different colors are clearly separated with minimal overlap, with combined loss achieving the best clustering performance.

To further investigate the effectiveness of fine-tuning, Figure 7 shows the label distribution in PCA-reduced space for RoBERTa-based approaches. Compared to the pre-trained baseline, fine-tuned models show improved clustering structure. RoBERTa-Center reduces intra-class distances but may merge distinct classes. RoBERTa-Cross maintains reasonable class separation but with less compact clusters. RoBERTa-Combined achieves the best balance, creating compact within-class clusters while preserving between-class boundaries. This raises the question of what the model learns to focus on during fine-tuning.

**Figure 8:**
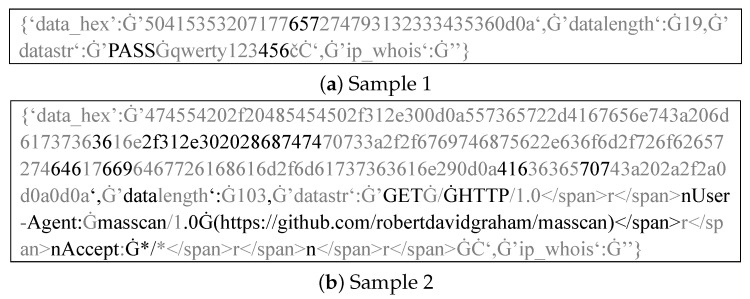
Visualization of Attention Focus in Cyber Bot Traffic Logs: Black regions indicate the model’s areas of focus. The symbol “Ġ” denotes a space character in RoBERTa’s tokenizer.

**RoBERTa-Combined specifically focuses on the content of the payload patterns section.** We leverage the attention mechanism in the RoBERTa model to compute and observe the attention distribution across tokens in the input text, providing an explanation for the payload pattern generation process, which is beyond the capabilities of traditional NLP techniques [25]. Figure 8 shows two examples of attention distribution in the logs, where tokens receiving the top 20% attention are shown in black and the remaining 80% in gray. We observe that the model focuses more on content reflecting the techniques or tools used by cyber bots, while ignoring less relevant information, such as password lists or randomly generated data. This allows payloads with similar cyber bot techniques to have more similar embeddings.

### 6.2. Application to Unlabeled Data

Having validated the embedding and clustering components and confirmed that the model’s attention can effectively distinguish payload patterns, we now apply TrafficPrint with the fine-tuned RoBERTa-Combined model from the previous subsection to the 21,379,802 unlabeled payloads. We use HDBSCAN clustering with min_cluster_size∈{10, 15, 20, 25, 30, 35} and min_samples∈{3, 5, 7, 10}, and select the final values of min_cluster_size=20 and min_samples=5 based on grid search over these discrete values, balancing cluster coverage and silhouette score. The resulting clusters are stable, with silhouette scores ranging from 0.39 to 0.60, indicating that the framework is relatively insensitive to small variations within this range and that the cluster structure remains stable. For pattern generalization, we apply agglomerative clustering with θ∈{0.6, 0.7, 0.8}, selecting θ=0.7 based on the coverage of the extracted patterns over the unlabeled payloads, which also remains robust across the tested range. Using TrafficPrint, we successfully extract 296 distinct payload patterns from the unlabeled data, which match 83.57% of the previously unknown entries (99.2% of the payloads), effectively enabling the automatic labeling of this large-scale dataset. Unlike detection systems evaluated by false positive rates on ground truth, our payload patterns themselves serve as labels derived directly from payload semantic content through automatic clustering and pattern extraction, greatly reducing manual effort. Each pattern represents a semantic cluster, meaning matched payloads are inherently analyzable rather than subject to classification errors. The practical benefit is quantified through coverage: our 296 automatically generated patterns expand the proportion of Honeypot payloads that can be systematically analyzed from 0.8% (covered by existing rule sets) to 83.7%, providing a long-term capability enhancement for behavioral analysis.

Furthermore, we compare the extracted payload patterns with the content sections (which search for specific content in packet data, closely resembling our payload patterns) of existing Snort and Suricata rule sets [56]. The collision rate is 0.67%, with only two patterns overlapping. The EHLO * pattern collides with SMTP reconnaissance detection rules such as abnormally large SMTP EHLO alerts, while *scanner* exhibits lexical overlap due to adversary-controlled fields such as domain names and user-agent strings containing the term *scanner* in security scanning services. This low collision rate indicates that the extracted payload patterns can complement the limitations of manual rules in recognizing emerging behaviors. These 296 patterns form the foundation for our behavioral analysis in Section 7, where we examine what they reveal about cyber bot operations in the wild, further validating the quality and diversity of the extracted patterns through their operational significance.

## 7. Mapping Cyber Bot Behaviors in the Wild

Prior work on cyber bot behavior analysis achieves limited coverage (typically 1–60% on their respective datasets [18,21,22,38,57]), precluding comprehensive characterization of cyber bot behaviors at Internet scale. We achieve 83.7% overall coverage (0.8% initially labeled by existing rules, plus 82.9% by our 296 extracted patterns), enabling the systematic analysis presented in this section. We examine cyber bot behaviors across both the initially labeled payloads and the 296 payload patterns extracted in Section 6.2, exploring the generation principles, identities, techniques, and strategies employed by cyber bots, contributing valuable insights to threat analysis [10]. In particular, we aim to answer the following research questions:

- RQ1: How are the behaviors generated by cyber bots?
- RQ2: Who is behind the cyber bot activities, and how is this identity conveyed through payloads?
- RQ3: What are the strategies employed by cyber bots, and how are they reflected in their behaviors?

### 7.1. Generation Principles (RQ1)

Payloads serve as the carriers of cyber bot behaviors, and the way in which cyber bots construct these payloads fundamentally drives their operational logic. To better understand the underlying generation principles, we examine the structure and content of payloads and categorize them into three types based on their degree of customization: fully customized payloads (11% of patterns), semi-customized payloads (82% of patterns), and default payloads (7% of patterns). The degree of customization reveals different approaches to payload construction, allowing us to understand the principles underlying cyber bot behavior generation.

**Fully customized payloads.** These payloads, with the highest level of customization, are not based on any publicly recognized protocols and are specifically crafted for cyber bot activities. Through our payload pattern analysis, we observe that these payloads are frequently employed in generic probing activities rather than being tailored to interact with specific services or ports. The following are two representative examples of fully customized payloads:

- MGLNDD_<IP><PORT>: A payload associated with the Magellan (MGLN) project [58] from RIPE Atlas [59], used for global connectivity measurement and reachability probing [60].
- LIOR*UP*ZZZZZZZZZ…ZZ: A non-standard payload with human-like filler text, appearing across various ports, likely used in automated probing to test service vulnerabilities or identify exposed resources [61].

**Semi-customized payloads.** These payloads are crafted within the constraints of specific service protocols, often utilizing a predefined set of patterns. By combining standard elements with custom modifications based on the adversary’s objectives, these payloads introduce a significant degree of unpredictability in bot behaviors. Their ability to blend within normal protocol structures while incorporating variable, customized content makes them more difficult to detect and analyze.

In HTTP service-related traffic, we observe that many HOST field contents include external links (see Table 5). Through analyzing these HOST fields, converting IP addresses to domain names via WHOIS lookups when necessary, we identify 5615 distinct domain names, with 1564 (27.9%) ranked among the top 1M domains according to Tranco [62]. This suggests that adversaries select domains from popular libraries to ensure non-empty URLs [63] or probe servers of popular websites to uncover hidden services or match server fingerprints. Less popular domains can serve as indicators of the organizations controlling cyber bots, since only specific entities are likely to access these obscure destinations. Additionally, in some HTTP-related CONNECT payload patterns, we find Base64-encoded identifiers that embed the request’s current timestamp. This suggests that these requests are likely generated by cyber bots that log or track scanning activity, possibly for replay or monitoring purposes [20].

**Default payloads.** Beyond customized payloads, cyber bot activities frequently involve the use of default example payloads. These payloads, which are often based on publicly available templates or service-specific default configurations, allow cyber bots to interact with services in a standardized way. This behavior is typically observed when cyber bots engage in routine checks or simple verifications of service availability. Rather than requiring custom payloads, bots use these predefined templates to test service responsiveness, ensuring the targeted service remains operational for further exploitation. As shown in Table 6, various default payloads are employed by bots across different services to conduct such basic functionality checks.

**Figure 9:**
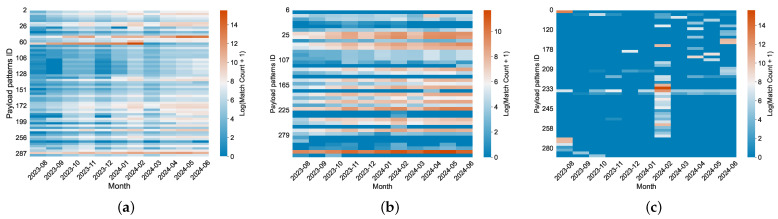
Heatmap of payload pattern match counts across months (ln scale), showing different patterns of cyber bot behavior: (**a**) patterns with rhythm match counts, (**b**) stable patterns, and (**c**) patterns with peak match counts in specific months.

**Temporal Modes.** Although the generation principles of behaviors can be explained through payload structure, important information such as the timing and frequency of occurrences also plays a key role. We discover three time-dependent modes in 54% of the payload patterns, illustrated through separate heatmaps. As shown in Figure 9a, we find that payload patterns (22%) show a matching trend consistent with the overall traffic volume for months, contributing to the traffic surge. Additionally, as shown in Figure 9b, payload patterns (14%) demonstrate a weak temporal correlation (e.g., ZG\x00\x00\...x0e\x01\x00), likely indicating simple scanning activities conducted by a single organization within a specific cycle. Moreover, payload patterns (18%) exhibit a sharp increase in traffic during specific months (Figure 9c). For instance, between February 4th and 22nd, the payload pattern CONNECT *r:443 HTTP/1.1\r\nHost: www.guzel.net.tr:443\r\n Connection:keep-alive\r\nUser-Agent: Mozilla/5.0 (Windows NT 10.0) AppleWebKit/537.36 (KHTML, like Gecko) Chrome/117.0.0.0 Safari/537.36\r\n\r\n appeared in large volumes, originating from servers like tube-hosting.com. These requests predominantly involved hosts conducting CONNECT tests on HTTP services, targeting cloud service providers and proxy servers.

### 7.2. Identities (RQ2)

The identity behind cyber bot behavior can be inferred from the payloads. Among the 296 extracted payload patterns, 39 patterns (13%) reveal identity-related information. While not representing all payloads, these identity-revealing patterns provide valuable insights into the identities of cyber bots, including information about the organizations and tools. We analyze identity information based on the degree of disclosure, examining both explicit declarations and implicit revelations embedded in the payloads.

**Explicitly.** Cyber bots often assert their identity through the use of User-Agent strings (See Table 7) and client identifiers embedded in their payloads. This is particularly evident in protocols such as MQTT, SMTP, and RDP, where bots declare their presence via specific identifiers. For instance, in MQTT, a variety of identifiers associated with services like CENSYS and LZR [67] are commonly observed. Similarly, in SMTP, cyber bots use the EHLO command to introduce themselves and query service extensions. Our analysis shows frequent use of tools like masscan and platforms such as www.censys.io, indicating bots leverage these services to probe SMTP servers. Table 8 presents the most common client identifiers observed. Additionally, some payloads feature empty or randomized identifiers, which do not declare a specific identity but are part of routine bot operations. On occasion, we observe payloads declaring themselves as legitimate Windows identifiers, such as win-clj1b0gq6jp.domain. Further details regarding RDP-related client identifiers can be found in Table 9. Notably, across all services, only 2 payload patterns from academic organizations included contact information and explicitly stated in the payload that users could make contact if they did not wish to be scanned. This provides users (i.e., scan targets) with an option to opt out.

**Implicitly.** In cases where cyber bots do not explicitly declare their identity, we infer it from pre-configured scripts, customized payloads (as discussed in Section 7.1), and organizational usage patterns.

Cyber bots often rely on pre-configured scripts for their operations. When performing CRUD operations on databases, bots typically use specific object names, such as yongger2 in Table 10. Similarly, when connecting to external servers to download malicious code, these payload patterns include external links that reveal server IPs, domains, or file paths, as seen in Table 10 (e.g., http://103.x.x.x/mps). These script behaviors serve as indicators of the cyber bot’s identity, as bots with similar configurations may share technical relationships. By recognizing these shared traits, defenders can shift from tracking individual IP addresses to defending groups of bots that exhibit similar behaviors.

Moreover, cyberspace search engines, such as Shodan, Censys, ZoomEye, and BinaryEdge, display distinctive cyber bot techniques. By analyzing the relative frequency of payload patterns within each platform, as summarized in Table 11, we identify their distinct underlying strategies. Notably, Shodan, ZoomEye, and BinaryEdge all demonstrate some unexpected instances of brute-force attacks. Shodan particularly emphasizes encrypted communication protocols, exploring security policies like STARTTLS and AUTH TLS. In contrast, BinaryEdge relies heavily on Nmap, generating payloads derived from Nmap’s built-in features, including GET /nice%20ports%2C/Tri%6Eity.txt%2ebak HTTP/1.0 and sip:nm SIP/2.0\r\nVia:.... Interestingly, only Censys employs the payload pattern *1\r$11\rNONEXISTENT\n, highlighting its distinctive approach to querying and identifying targets.

**Figure 10:**
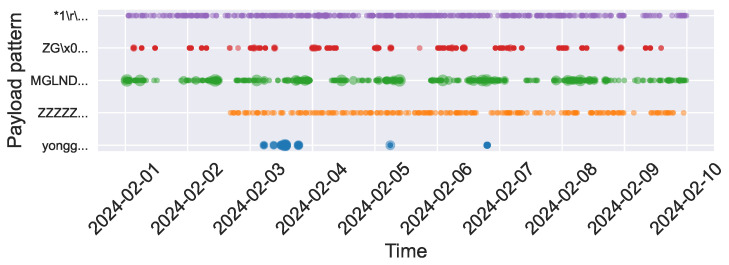
Periodicity of payload patterns implicitly revealing identities. This is a bubble chart, where larger points indicate higher counts and denser areas indicate more frequent occurrences.

Based on the analysis above, we select payload patterns containing pre-configured file information, customized payloads, and those associated with specific organizations to examine periodic trends during the top 10 traffic days in February. As shown in Figure 10, these payload patterns, which implicitly reveal cyber bot identities, exhibit clear periodicity and distinct behavioral patterns. These identity-specific behavioral signatures enable attribution beyond IP-based tracking: when similar payload sequences appear from new sources, defenders can cluster them as likely coordinated infrastructure based on shared configuration fingerprints [9,22], even when source addresses rotate. Within a 10-day observation window, the three pattern categories demonstrate distinguishable temporal characteristics. Organization-associated patterns like Censys (NONEXISTENT, purple curve) maintain consistent daily reconnaissance activities. Customized payloads exhibit varied rhythms: some show intermittent activity within specific cycles (ZG..., red curve), while others display persistent high-frequency scanning (MGLNDD, ZZZZZ, green and orange curves). Pre-configured scripts (yongger, blue curve) demonstrate sporadic bursts characteristic of event-driven operations. These temporal patterns map to distinct cyber bot operational lifecycles, ranging from continuous reconnaissance to opportunistic exploitation phases. Such temporal behavioral signatures enable organization attribution [38] and cyber bot type classification within short observation periods.

### 7.3. Strategies (RQ3)

**Figure 11:**
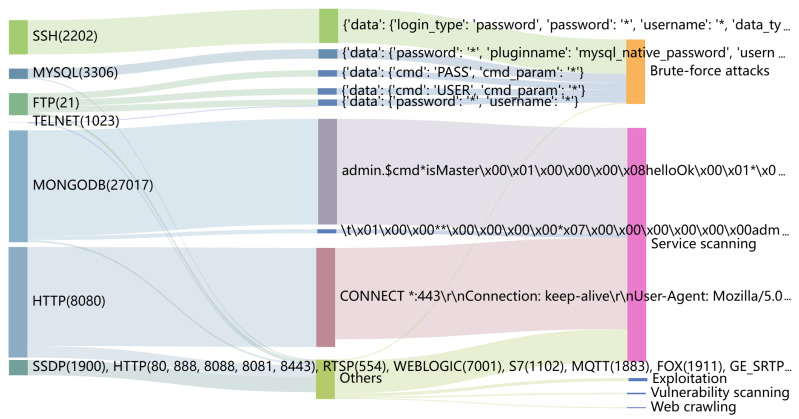
Flow of cyber bot traffic from ports to five behavioral categories, with arrow width indicating the contribution from each source.

We identify distinct cyber bot strategies by analyzing the extracted payload patterns. Through careful observation of both payload pattern content and associated traffic log metadata (such as protocol, port, IP, and timestamps) combined with domain expertise in cybersecurity, we classify these behaviors into five distinct categories. Our classification is derived from recognizable attack signatures, command structures, and interaction patterns observed across the dataset. Table 1 defines and distinguishes the behaviors associated with these payload patterns. Among the labeled payloads, which are obtained using both public rules and TrafficPrint, the distribution across behavior categories is as follows: service scanning (78.14%), Web crawlers (0.39%), brute-force attacks (19.98%), vulnerability scanning (0.62%), and exploitation (0.86%). Figure 11 visualizes the flow of cyber bot traffic, with the ports displayed on the left. The traffic corresponding to the payload patterns received by the honeypot’s forged services is then categorized according to specific cyber bot behaviors, where the width of the arrows indicates the contribution from each category. Analyzing different cyber bot behaviors provides insights into their strategic preferences, such as selecting specific services or adapting payload content to evade detection.

**Service Scanning.** The deployment of a multi-service honeypot significantly broadens the scope of cyber bot activities, leading to a wider variety of behaviors. Consequently, service scanning represents a substantial portion of the observed traffic.

Non-standard ports are commonly targeted in scans. The mstshash parameter, associated with the Microsoft Remote Desktop Protocol (RDP) cookie, frequently appears in payloads. Table 9 lists the top 10 most common values for this parameter, which include valid usernames used by servers for client identification, load balancing, and session management [68]. We observe a high volume of such payloads from cyber bots targeting the standard RDP port (3389). Notably, similar payloads are also found on a range of non-standard ports. This indicates that while developers recognize the prevalence of non-standard ports, adversaries are also adapting their scanning techniques to target these ports [67].

**Web Crawling.** This behavior typically involves sending HTTP GET requests to retrieve web pages and parsing HTML content for links, text, and metadata. In our honeypot, we serve static interfaces rather than real website templates, limiting the effectiveness of metadata crawling. However, we observe generic reconnaissance behaviors, such as frequent requests for robots.txt and /sitemap.xml, which account for 35,961 out of 841,578 total HTTP requests (see Table 12).

Malicious cyber bots often disguise themselves as legitimate web crawlers, like Googlebot or Bingbot, by spoofing the User-Agent field (Table 7). While search engine crawlers help users by indexing accessible links, they also provide attackers with a means to access sensitive resources undetected. For example, bots masquerading as msnbot attempt to access sensitive files, such as /systembc/password.php, indicating an attempt to exploit vulnerabilities. This underscores that in behavior detection, a bot’s observable actions are more reliable indicators than its declared identity, which may be spoofed.

**Brute-force Attacks.** Brute-force attacks systematically try all possible combinations to guess authentication credentials, relying on computational power, which leads cyber bots to employ targeted strategies to optimize the process.

**Figure 12:**
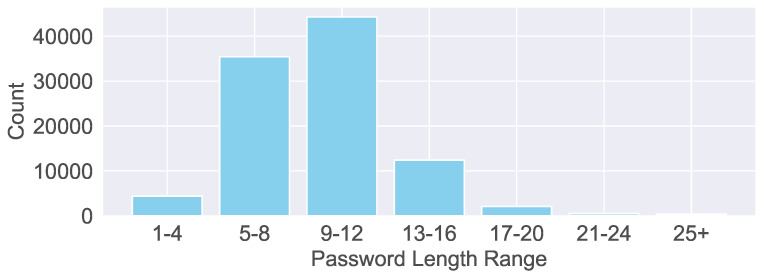
Distribution of password lengths used in brute-force attacks, showing the frequency of each password length in the collected traffic data.

Weak passwords continue to be a primary focus within password dictionaries. Figure 12 illustrates the distribution of password lengths used by cyber bots, with the majority falling between 9 and 12 characters. In contrast, credentials longer than 20 characters are generally considered more secure. In terms of password complexity, we observe the following distribution: low complexity (41.86%), consisting solely of letters; medium complexity (50.62%), incorporating both letters and numbers; and high complexity (7.53%), combining uppercase and lowercase letters, numbers, and special characters.

**Figure 13:**
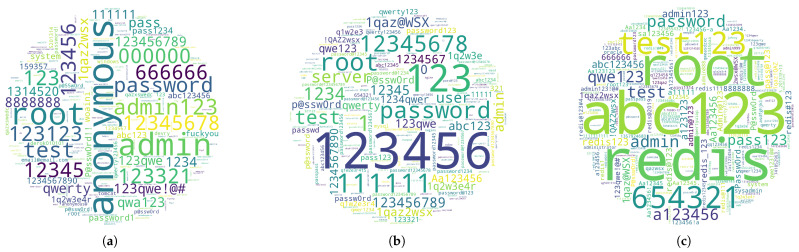
Wordcloud distribution of passwords used in brute-force attacks against different services (Word size indicates password frequency). (**a**) FTP service; (**b**) SSH service; (**c**) REDIS service.

Cyber bots tailor their password sets to the specific service. Figure 13 shows the passwords attempted on different services, demonstrating service-specific trends. For example, FTP servers often experience numerous login attempts with anonymous as the password, while Redis servers see frequent use of redis. The most common passwords for each service typically align with the legitimate default credentials. Adversaries regularly update and refine their dictionaries, making repeated attempts on compromised services.

Most cyber bots’ brute-force strategies remain unaffected by the password policy configurations of the targeted services. In classic versions of Redis, authentication relies solely on a password, without the need for usernames. Users only need to provide the password via the AUTH command upon connection. Our Redis instance intentionally omits a password setting, yet we still record a high volume of AUTH attempts. This indicates that cyber bots generally do not tailor their brute-force routines to the actual security configuration of a service, but instead follow a fixed authentication pattern regardless of whether password protection is enabled.

Different adversaries employ varied brute-force strategies. Some brute-force attempts include non-password commands, such as the INFO command in Redis or the QUIT command in FTP. This suggests that adversaries not only rely on password combinations but also issue system commands to gather additional information about the target system or terminate connections at specific points to evade detection. This hybrid strategy creates multidimensional attack vectors that challenge conventional defense mechanisms. Thus, analyzing the usage of these non-password commands can provide valuable insights into the attacker’s tactics, Techniques, and Procedures (TTPs), which can subsequently improve the specificity and effectiveness of defense measures.

**Vulnerability Scanning.** Vulnerability scanning builds on service scanning by querying for non-destructive defects once a service protocol is identified. As shown in Table 13, cyber bots methodically target sensitive resources over HTTP services. Common targets include configuration files, such as .env, which may expose credentials and system keys, and .git/config files, revealing source code and version control histories. Misconfigurations in application servers, like Tomcat, can lead to unauthorized access, while paths like /rediss.php?i=id and /geoserver/web/ expose further attack vectors. Therefore, proper isolation of environments by function (development, testing, and production), encryption, access controls, and routine audits are essential to mitigate these risks.

**Exploitation.** Exploitation targets specific vulnerabilities, often requiring manual effort, but cyber bots can exploit common weaknesses with low-cost attacks, such as unauthorized access, interacting with external resources, or executing malicious commands like database deletion. From the number of log entries matching the payload patterns corresponding to exploitation behaviors, we obtain the most commonly used commands and scripts employed by cyber bots. Table 10 lists these representative payloads.

Encoding-based evasion techniques, especially base64, are widely adopted by advanced cyber bots to conceal malicious intent and bypass basic defenses. Complex cyber bots often employ techniques aimed at bypassing basic signature-based defenses. We observe that adversaries convert executable files in Windows into hexadecimal format and insert them into SQL injection payloads, such as \x17\x1c\x00\x00\x03 set@a = concat(”,0x4D5A...0000). Additionally, they may encode malicious commands (e.g., curl, killall) or scripts from remote servers (e.g., //95.x.x.x:3306 /Tomcat Bypass/Command/Base64/Y3VybCAtcyA...) using base64 encoding, both to avoid encoding issues and to obscure their malicious intent. In the payload patterns that employed evasion techniques, 80% are encoded in base64.

## 8. Conclusions and Future Work

This paper conducts an Internet measurement using TrafficPrint, a framework for automated extraction of payload patterns and labeling of cyber bot behaviors. Over 11 months, we collected traffic from 8 regions and generated 296 patterns, covering 83.57% of unknown payloads. These patterns reveal emerging threats beyond traditional IDS coverage and offer insights into why many behaviors evade detection, supporting improved defense strategies.

Although TrafficPrint effectively captures and generalizes unknown payload behaviors, several aspects merit further exploration. The current framework focuses on interpretable and scalable pattern extraction from payload content, which inevitably trades off some sensitivity to heavily obfuscated or encrypted behaviors, which account for a large portion of the remaining 16.43% of unlabeled traffic. Future work will enhance TrafficPrint with richer cooperative behavioral features and temporal modeling to better characterize these residual unknowns and enhance the generalization of extracted patterns across diverse network environments.

## Figures and Tables

**Table 1:** Different cyber bot behaviors definitions and key characteristics.

Behavior	Cyber Bots	Definition
Service Scanning	Nmap, Masscan	Systematically identifying open ports and available services.
Web Crawling	Googlebot	Collecting web content by following known URLs, such as ‘robots.txt’.
Brute-force Attack	Hydra, Burp Suite	Attempting authentication repeatedly, using static or dynamic credentials.
Vulnerability Scanning	Nessus, Nuclei	Detecting security weaknesses in identified services using crafted requests.
Exploitation	Metasploit	Gaining unauthorized control by leveraging vulnerabilities or exploit code.

**Table 2:** Distribution of Traffic Logs and L7 Payloads Across Top-5 Protocols.

Protocol	Traffic Logs		L7 Payloads		Payload Rate (%)
	Count	%	Count	%	
HTTP	17,847,129	25.12	5,251,734	24.37	29.43
MongoDB	16,918,870	23.81	4,818,028	22.36	28.48
MySQL	7,004,033	9.86	721,098	3.35	10.30
SSH	4,953,637	6.97	1,989,321	9.23	40.15
FTP	2,151,656	3.03	1,272,897	5.91	59.16
Others	22,172,209	31.21	7,495,175	34.78	33.81
Total	71,047,534	100.00	21,548,253	100.00	30.33

**Table 3:** Comparison of Purity, F1, FMI, and ARI for different methods.

Method	Purity	F1	FMI	ARI
Word2Vec	0.9389	0.8366	0.7927	0.7095
RoBERTa-Base	0.8899	0.7714	0.7405	0.6391
RoBERTa-Center	0.9506	0.8941	0.9499	0.9288
RoBERTa-Cross	0.9996	0.9996	0.9995	0.9993
RoBERTa-Combined	0.9998	0.9998	0.9998	0.9997

**Table 4:** Sensitivity Analysis of λ on RoBERTa-Combined with different training data sizes.

Training Data	λ	Purity	F1	FMI	ARI
20% of full training set	0	0.8674	0.8440	0.8465	0.8540
	0.1	0.8694	0.8586	0.8475	0.8623
	0.3	0.8976	0.8846	0.8553	0.8892
	0.5	0.9304	0.9417	0.9401	0.9577
	0.7	0.9263	0.9357	0.9364	0.9399
	0.9	0.9132	0.9215	0.9267	0.9304
	1	0.9146	0.9264	0.9286	0.9284
50% of full training set	0	0.9090	0.9025	0.8980	0.9270
	0.1	0.9649	0.9453	0.9571	0.9688
	0.3	0.9621	0.9612	0.9610	0.9832
	0.5	0.9884	0.9880	0.9873	0.9907
	0.7	0.9867	0.9893	0.9752	0.9841
	0.9	0.9748	0.9737	0.9715	0.9781
	1	0.9713	0.9699	0.9686	0.9707

**Table 5:** Top 7 outbound HTTP requests.

Domain	Requests	Unique IPs	Top-1m Rank
castores.com.mx	840,751	10	299,936
guzel.net.tr	592,949	18	40,709
olimpbet.net	548,415	19	/
rma.polizei.hessen.de	481,204	1	/
darinskzelrb.kz	425,991	18	/
olimpcom.kz	345,924	1	/
bj38.live	232,267	1	/

**Table 6:** Payloads of official default examples (sources: UPnP [64], Redis [65], RFC 4217 [66]).

Payload	
M-SEARCH * HTTP/1.1\r\nHost:239.255.255.250:1900\r\nST:ssdp: all\r\n Man:“ssdp:discover”\r\n MX:3\r\n\r\n	*1\r\n$4\r\nQUIT\r\n AUTH TLS\r\n

**Table 7:** Top 14 Web Crawler User-Agents in HTTP requests.

User Agent	Requests	User Agent	Requests
GenomeCrawlerd	2596	grub-client	23
Googlebot	1938	YandexBot	18
AdsBot-Google-Mobile	1029	archive.org_bot	16
bingbot	266	TurnitinBot	10
QBOT	56	Amazonbot	10
msnbot	33	Twitterbot	8
Nimbostratus-Bot	26	FAST-WebCrawler	7

**Table 8:** SMTP Client Identifiers in EHLO Commands.

Client Identifier for EHLO Command		
masscan	leakix.net	(empty)
DESKTOP-CVJNS4K	ADMIN	scanner.sslsonar.org
www.censys.io	openssl.client.net	localhost
User	hello	win-clj1b0gq6jp.domain
mail.example.com	scanworker-Linux	ABC

**Table 9:** Top 10 most frequent contents in the ‘Cookie: mstshash=*’ payload pattern.

Cookie: Mstshash=*	
Administr	nmap
Administrator	beio
\x01\x00\x08\x00\x01\x00\x00\x00	hello
Test	Domain
eltons	yhDxyQONx

**Table 10:** Typical exploitation commands observed in Redis, MySQL, and HTTP services (simplified for presentation).

Redis	MYSQL		HTTP
NONEXISTENT	show variables like ‘%version_compile_os%’		POST /goform/set_LimitClient_cfg HTTP/1.1\r\nCookie: user=admin\r\n\r\ntime1=00:00- 00:00\&time2=00:00-00:00\&mac=;wget http://103.x.x.x/mpsl; chmod 777 mpsl; ./mpsl lblink.selfrep;\r\n\r\n
INFO	SHOW DATABASES	CREATE *	
config *	commit *	service iptables stop	
CLIENT	drop *	insert into yongger2 values(“”)	
flushall	FLUSH PRIVILEGES	update yongger2 set data = @a	
SET *	show global variables like ‘secure_file_priv’		
Eval *	SET *	SELECT VERSION()	

**Table 11:** Payload patterns favored by different cyberspace search engines (simplified to show key content), ordered by descending frequency within each engine.

Shodan	Censys	ZoomEye	BinaryEdge
\x08http/0.9\x08http/1.0\x08 http/1.1\x06spdy/1\x06spdy	GET/HTTP/1.1\r\n	\x08http/1.1\x06spdy/1\x06 spdy/2\x06spdy	\x00\x00\x00admin.$cmd\x00...
EHLO *	Mozilla/5.0 (compatible; CensysInspect/1.1; +https://about.censys.io/)	GET /robots.txt HTTP/1.1\r\n	\x2ACookie: mstshash=
STARTTLS\r\n	GET /favicon.ico HTTP/1.1\r\n	GET /favicon.ico HTTP/1.1\r\n	GET/HTTP/1.0\r\n\r\n
AUTH TLS	PRI * HTTP/2.0\r\n\r\nSM \r\n\r\n\	*1\r$4\rINFO\r\n	\x08http/0.9\x08http/1.0\x08h ttp/1.1\x06spdy/1\x06spdy
GET /favicon.ico HTTP/1.1\r\n	*1\r$4\rPING\r\n	OPTIONS/RTSP/1.0\r\n\r\n	\x10\x2A\x00\x04MQTT\x04
*1\r$4\rINFO\r\n	*1\r$11\rNONEXISTENT\r\n	USER *	GET /nice%20ports%2C/Tri%6Eity .txt%2ebak HTTP/1.0\r\n\r\n
HELP\r\n	*1\r$4\rQUIT\r\n	PASS *	sip:nm SIP/2.0\rVia:...

**Table 12:** Top 5 paths accessed by crawler.

Rank	Path	Requests
3	/favicon.ico	35,961
9	/robots.txt	10,750
10	/sitemap.xml	9028
72	/index.html	672
78	/form.html	653

**Table 13:** Top 10 paths accessed by cyber bots in vulnerability scanning.

Paths	
/.env	/_profiler/phpinfo
/manager/html	/server-status
/config	/geoserver/web/
/rediss.php?i=id	/.git/config
/stacks	/jars

## Data Availability

The datasets presented in this article are not readily available because the data are derived from continuously operating honeypot systems that contain sensitive security information. Public disclosure of these datasets would compromise the operational effectiveness of our honeypot infrastructure for ongoing threat detection and analysis, and could enable adversaries to identify or evade our monitoring systems. Requests to access the datasets should be directed to the corresponding author; however, due to the sensitive nature of the data and ongoing security operations, access cannot be granted.
